# Implementing Transitional Care Interventions for Surgical Patients: A Scoping Review

**DOI:** 10.1111/jan.70081

**Published:** 2025-07-21

**Authors:** G. Tobiano, B. M. Gillespie, K. Turner, A. M. Eskes, B. Patel, J. Colquhoun, L. Ferronato, W. Chaboyer

**Affiliations:** ^1^ NHMRC Centre of Research Excellence in Wiser Wound Care Griffith University Southport Queensland Australia; ^2^ Gold Coast University Hospital, Gold Coast Hospital and Health Service Southport Queensland Australia; ^3^ School of Nursing and Midwifery, Griffith University, Gold Coast Campus Southport Queensland Australia; ^4^ Griffith University, Gold Coast Campus Southport Queensland Australia; ^5^ Department of Surgery Amsterdam UMC Amsterdam the Netherlands; ^6^ Princess Alexandra Hospital Brisbane Queensland Australia; ^7^ Bond University Robina Queensland Australia

**Keywords:** continuity of patient care, hospital to home transition, inpatients, nurses, operative, patient discharge, postoperative care, review, surgical procedures, transitional care

## Abstract

**Aims:**

To synthesise the evidence on implementation strategies used to implement transitional care interventions for adult surgical patients.

**Design:**

Scoping review.

**Data Sources:**

Medline, CINAHL and EMBASE were searched in August 2023 and updated June 2025, followed by citation searches. Studies were screened independently by two researchers, and one extracted data, another verified its accuracy. Studies about transitional care interventions for adult surgical patients were coded according to the ‘Five classes of implementation strategies’ and the ‘Patterns, Advances, Gaps, Evidence for practice and Research recommendations’ framework, to illuminate the review findings.

**Results:**

Based on 27 studies included in the scoping review, staff education, changes to staffing and electronic systems, and change management techniques were frequently used implementation strategies. Implementation strategies were mostly used with patients undergoing colorectal and cardiac surgery in Asia and the United States. Scale‐up strategies and capacity‐building initiatives for people in charge of spearheading the change initiatives were less common.

**Conclusions:**

To further the field, future research could focus on capacity‐building and scale‐up strategies, fidelity reporting, and financial implications of implementation in a wider range of surgical populations and settings. Work is needed to effectively implement surgical transitional care interventions in real‐world settings.

**Implication for the Profession and/or Patient Care:**

Our findings provide strategies for hospital leaders to adopt when implementing transitional care interventions for surgical patients.

**Reporting Method:**

Scoping Reviews (PRISMA‐ScR) checklist.

**Patient or Public Contribution:**

Determined review focus, interpreted findings, and contributed to manuscript.

**Review Registration:**

The Open Science Framework.

**Summary**

- Provides key tactics for hospital staff when implementing surgical transitional care interventions, and clear gaps for future researchers such as testing scale‐up strategies.

## 1 Introduction

Almost 30% of patients who have undergone major surgery have postoperative complications after hospital discharge (Morris et al. 2014). Of these complications, 59% are associated with hospital readmission (Morris et al. 2014) and cost the US healthcare systems $269 million (Brown et al. 2021). There has been growing economic pressure on hospitals to contain costs, with fast‐track protocols being implemented to reduce patient length of stay (Fiona 2014). However, transitioning home quicker may increase patient and caregiver burden if they are not adequately supported to manage the postoperative recovery (Eskes et al. 2023). Common challenges patients and caregivers face include difficulty recognising complications and uncertainty about when and where to seek help, often leading to unnecessary emergency department visits (Kang et al. 2020). Patient education and follow‐up in the community have been suggested to support better postoperative complication management and, in turn, reduce hospital readmissions (Jones et al. 2016). Ultimately, targeted strategies are needed to enhance postoperative care to reduce readmissions for surgical patients.

## 2 Background

Transitional care interventions (TCIs) are interventions utilised to ensure continuity of care as patients move across healthcare settings (Naylor et al. 2011). They do not have a specific event that denotes their start and end point; they start in hospital and continue after discharge, to support patients and their caregivers to manage recovery (Holland and Harris 2007). TCIs are multi‐component, which is defined as an approach incorporating many strategies to tackle a specific issue, such as behavioural, educational and organisational techniques (Craig et al. 2008). Common components of TCIs include engaging patients and caregivers in care plan development, education and self‐management, and ensuring seamless contact between patients/caregivers and healthcare professionals in the community (Hirschman et al. 2015). One of the most studied and effective TCI models is the Naylor model, which incorporates these components but is primarily applied to older medical patients with multiple chronic conditions (Hirschman et al. 2015). Surgical patients face unique risks, particularly postoperative complications associated with the anaesthetic, surgical procedure and subsequent incision or wound, highlighting the need for TCIs specifically tailored to their needs.

TCIs are proposed to promote surgical patient recovery by enhancing activities of daily living and quality of life, and reducing emergency department visits and hospital readmission rates (Mao et al. 2022). For example, a review found that surgical patients who receive TCIs were 40% less likely to be readmitted to hospital (Mao et al. 2022). TCIs may also positively influence surgical patient perceptions of their transition experience and satisfaction with care received (Tobiano et al. 2025). Evidence suggests that TCIs with more intervention components tend to show more positive outcomes (Tobiano et al. 2025). Despite their potential, TCIs for surgical patients remain an underutilised strategy, presenting a solution waiting to be realised.

However, TCIs are complex interventions, and those implementing TCIs must navigate the inherent tensions of using them in the real world (Fakha et al. 2021). Implementation research focuses on identifying barriers and facilitators to intervention uptake, and identifying implementation strategies to address these barriers and promote the facilitators to ultimately increase intervention adoption (Bauer and Kirchner 2020). The complexity of implementation strategies lies in their need to address multiple factors concurrently, such as providing education, engaging stakeholders, securing resources and ensuring sustainability at the same time (Proctor et al. 2013). Previous reviews tend to overlook the importance of implementation for maximising impact. For example, in a review of TCIs across a range of settings, most researchers failed to report implementation strategies (Rennke et al. 2013). For reviews that are specific to surgical TCIs, most aim to determine intervention effectiveness rather than implementation effectiveness or implementation fidelity (Jones et al. 2016; Mao et al. 2022). Yet, a review on TCIs for older people showed that implementation strategies are critical at both the individual and organisational level to address common barriers such as lack of resources and higher priority for implementation of other initiatives (Fakha et al. 2021, 2023). Before implementing interventions into practice, a clear implementation plan for surgical TCIs is required to ensure intervention outcomes are achieved. Thus, this scoping review aimed to synthesise the evidence on implementation strategies used to implement TCIs for adult surgical patients. This scoping review is complementary to a published review of TCI components and outcomes (Tobiano et al. 2025).

## 3 Methods

### 3.1 Design

A scoping review was undertaken, and reported as per the Preferred Reporting Items for Systematic Reviews and Meta‐Analyses extension for Scoping Reviews (PRISMA‐ScR) Checklist (Tricco et al. 2018). The protocol was registered a priori (https://osf.io/kf2v7/). The stages for a scoping review were followed which include: (1) identifying the research question, (2) identifying relevant studies, (3) study selection, (4) charting the data, (5) collating, summarising and reporting the results and (6) consultation (Arksey and O'Malley 2005).

#### 3.1.1 Identifying the Research Question

The mnemonic ‘population, concept and context (PCC)’ was used to frame the review question (Peters 2015). The population was adult, hospital surgical patients. The concept was multi‐component TCIs, that occur across the intra‐ and post‐ hospital setting, such as patient education and follow‐up in the community (Holland and Harris 2007). The context was in hospital and post‐hospital, where post‐hospital care was being managed by the patient/caregiver. Thus, the review questions was: ‘What strategies are used to implement TCIs for adult surgical patients?’.

#### 3.1.2 Identifying Relevant Studies

Searches occurred in August 2023, and were updated in June 2025, in the computerised databases Medline, CINAHL and EMBASE. The search strategy used subject headings and search terms, informed by crucial articles in the field and health librarian input (See File S1). Examples of subject headings and search terms were ‘perioperative’, ‘surgical procedures, operative+’, ‘transition’ and ‘transitional care’. Additionally, studies that were included had their reference lists searched for past studies that met eligibility criteria. Finally, Scopus was used to find newer articles that had cited the included articles.

#### 3.1.3 Study Selection

Duplicates were removed in EndNote 20 and then again in Covidence systematic review software (www.covidence.org). Studies were screened independently by two researchers in the Covidence systematic review software package based on eligibility criteria.

Inclusion criteria were:

1. TCIs with ≥ 2 components, aimed at adult surgical inpatients, because it aligns with our phenomenon of interest.
2. ≥ 1 Intervention components delivered by a nurse, due to their pivotal role (Mao et al. 2022).
3. Reports implementation strategies, as this addresses our research question.
4. Original peer‐reviewed research and quality improvement projects, in English, published from 2016 to 2025. These criteria were selected to capture a wide range of TCIs, not just those evaluated by randomised controlled trials (Jones et al. 2016; Mao et al. 2022), in a language widely accessible and understood by the research team. Additionally, the date range reflects the release of the World Health Organisation's guidelines for enhancing care transitions (World Health Organization 2016).

Exclusion criteria included:

1. Interventions that include post‐hospital components occurring in rehabilitation settings or care facilities, where patients receive 24/7 care from healthcare professionals, due to this being an alternative care pathway.
2. Paediatrics, where data for adults cannot be disconnected, as paediatrics necessitate a different approach to TCIs.
3. Letters, commentaries, editorials, conference abstracts/presentations, protocols, theses, reviews, grey literature, because of absence of peer review and paucity of detail about implementation strategies.

First, researchers screened titles and abstracts, and those studies labelled ‘include’ or ‘maybe’ had full‐texts retrieved and were screened again. Disagreements were resolved through discussion and unresolved disagreements were adjudicated by a third researcher. Study selection is presented narratively and as a flow diagram (Tricco et al. 2018).

#### 3.1.4 Charting the Data

A data extraction form was designed by our team, which included author/year/country, setting, type of surgery, implementation fidelity (i.e., were implementation strategies used as intended) and intervention fidelity. We piloted the form with six studies and then one researcher extracted data, and a second confirmed its accuracy. A third researcher could arbitrate disagreements, but was not needed.

#### 3.1.5 Collating, Summarising and Reporting the Results

Data extraction tables were summarised narratively.

The first step of data synthesis was to identify patters. Thus, we returned to the original articles, and mapped the strategies used to implement TCIs as per Leeman et al.'s (2017) ‘Five classes of implementation strategies'. These classes include: (1) dissemination strategies defined as activities aimed at raising the understanding, knowledge, attitudes and intentions of staff to adopt the intervention; (2) implementation process strategies which are processes or actions done by the implementation team to plan, choose and integrate the intervention into practice; (3) integration strategies are any actions that address elements supporting or obstructing best possible integration of the intervention; (4) capacity‐building strategies aim to increase people’s overall ability to execute implementation process strategies (described in bullet point 2) and (5) scale‐up strategies are used to implement the intervention in multiple settings. Additionally, we identified who the target audience was for the implementation strategy and the person responsible for enacting the implementation strategy. These data were presented as a table.

Second, the ‘Patterns, Advances, Gaps, Evidence for practice and Research recommendations (PAGER) framework’ was used to synthesise implementation strategy patterns found (Bradbury‐Jones et al. 2021). This was reported in a table starting with Pattern (P), to present the patterns identified when summarising the implementation strategies as per Leeman et al.'s (2017) ‘Five classes of implementation strategies'. Under Advances (A), what the patterns could add into the current body of literature was reported. Based on the review results, the research Gap (G) column was used to identify knowledge gaps whereas Evidence of practice (E) reported suggestions for clinical practice. Lastly, Research recommendations (R) was used to recommend future research based on the previous four domains of the PAGER framework.

#### 3.1.6 Consultation

A health consumer who had experienced surgeries, a surgical ward nurse responsible for discharge planning, and a surgeon reviewed the patterns found and provided their interpretations, which shaped the PAGER findings, discussion and subsequent manuscript. Health consumer engagement is reported using the GRIPP2 reporting checklist (Staniszewska et al. 2017) (See File S2).

## 4 Results

**FIGURE 1:**
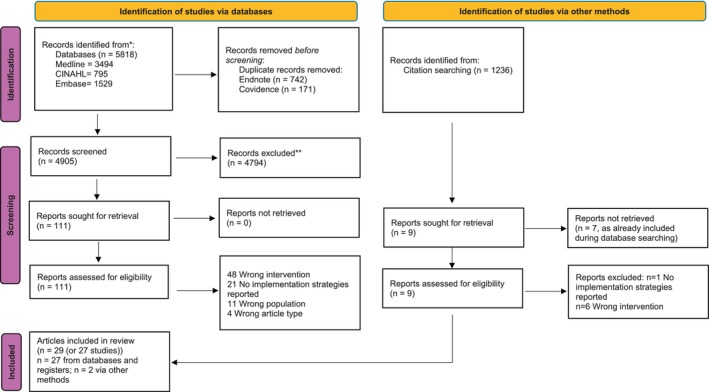
PRISMA flowchart.

In total 27 studies are included in our findings. The database search resulted in 5818 articles, and citation searching resulted in 1236 articles (See Figure 1). After removing duplicates and screening, 29 articles were included. The articles by Robertson et al. (2018) and Liu et al. (2019) and Zhang et al. (2020) and Zhang et al. (2021) reported on the same two studies and interventions, however, different outcomes were reported in each article, thus Robertson et al.'s (2018) and Liu et al.'s (2019) articles were counted as one ‘study’ and Zhang et al.'s (2020) and Zhang et al.'s (2021) articles were counted as one ‘study’ when reporting the findings.

### 4.1 Study Characteristics

See File S3 for data extraction. Studies were conducted in Asia (13/27 (48%)) (Zhang et al. 2020, 2021; Hu et al. 2020; Li et al. 2020, 2024; Lin et al. 2025, 2024; Tian et al. 2023; Tseng et al. 2021; Tu et al. 2024; Wang and Gao 2025; Xu 2021; Yang et al. 2023; Zhou et al. 2023), USA (12/27 (44%)) (Robertson et al. 2018; Liu et al. 2019; Aicher et al. 2019; Du 2021; Fisher et al. 2018; Fitz et al. 2020; Grahn et al. 2019; Iseler et al. 2018; Koeckert et al. 2017; Mitchell 2022; Pelt et al. 2018; Weintraub et al. 2018; Zuckerman 2020), Turkey (1/27 (4%)) (Coskun and Duygulu 2022) and Canada (1/27 (4%)) (Ahmadi et al. 2021). The surgical populations in the studies were colorectal and complex abdominal surgery (8/27 (30%)) (Zhang et al. 2020, 2021; Li et al. 2024; Lin et al. 2025, 2024; Zhou et al. 2023; Du 2021; Fisher et al. 2018; Grahn et al. 2019), cardiac (6/27 (22%)) (Tu et al. 2024; Aicher et al. 2019; Iseler et al. 2018; Koeckert et al. 2017; Weintraub et al. 2018; Coskun and Duygulu 2022), orthopaedic (4/27 (15%)) (Tseng et al. 2021; Xu 2021; Mitchell 2022; Pelt et al. 2018), neurological (3/27 (11%)) (Robertson et al. 2018; Liu et al. 2019; Yang et al. 2023; Zuckerman 2020), kidney (2/27 (7%)) (Hu et al. 2020; Li et al. 2020), lung (2/27 (7%)) (Fitz et al. 2020; Ahmadi et al. 2021), urology (1/27 (4%)) (Wang and Gao 2025), and gynaecological (1/27 (4%)) (Tian et al. 2023).

Implementation fidelity was most evident for Leeman et al.'s (2017) ‘implementation process’ strategies. ‘Selecting a change management process’ and ‘assessing the context’ were enacted by explicitly detailing the change management process selected (Xu 2021; Du 2021; Fitz et al. 2020; Mitchell 2022; Zuckerman 2020) and providing data of assessments conducted (Fisher et al. 2018; Fitz et al. 2020; Koeckert et al. 2017; Pelt et al. 2018; Zuckerman 2020). How well or not well ‘staffing changes and role clarification’ went was also frequently reported (5/27 (18.5%)) (Du 2021; Fisher et al. 2018; Iseler et al. 2018; Mitchell 2022; Zuckerman 2020). Additionally, evidence that staff had received training to support intervention delivery was reported in almost one in five studies (5/27 (18.5%)) (Li et al. 2020, 2024; Lin et al. 2025, 2024; Zhou et al. 2023).

Intervention fidelity was not reported in 16/27 (59%) studies. In the remaining 11/27 studies, intervention fidelity ranged from 20 to 100% for intervention components in 8/27 (30%) studies (Tseng et al. 2021; Yang et al. 2023; Du 2021; Fitz et al. 2020; Grahn et al. 2019; Iseler et al. 2018; Mitchell 2022; Zuckerman 2020), and 3/27 (11%) mentioned intervention fidelity but did not report specific results (Hu et al. 2020; Tu et al. 2024; Fisher et al. 2018).

### 4.2 Results of Data Mapping

The implementation strategies are in Table 1. They were most frequently categorised as ‘dissemination’, ‘integration’ or ‘implementation process’ strategies. The most frequent dissemination strategy was staff education and training to support intervention delivery. This differed from simply making staff aware of the intervention, but instead involved detailed training in areas like vascular medicines and surgery (Aicher et al. 2019), dehydration and acute kidney injury (Grahn et al. 2019), enterostomy skills (Zhang et al. 2020, 2021), education about clinical issues and community resources (Weintraub et al. 2018) and transitional care training inclusive of an exam (Li et al. 2020). The people responsible were not reported.

**TABLE 1:** Implementation strategies used as per Leeman et al.'s (2017) ‘Five classes of implementation strategies’.

Target audience	Person responsible (who enacted the strategy)	Strategy	# studies that used the strategy (# studies that mentioned person responsible)	References
**Dissemination strategies**				
Nurses, nurse managers, enterostomists, pharmacists, social workers, physicians, doctor kidney transplant surgeons, attending physicians, kidney transplant specialist nurses, transitional nursing staff	Not reported	Staff education and training to support the intervention delivery	11 (0)	(Hu et al. 2020; Li et al. 2020, 2024; Lin et al. 2025, 2024; Wang and Gao 2025; Zhou et al. 2023; Aicher et al. 2019; Fisher et al. 2018; Grahn et al. 2019; Weintraub et al. 2018)
Nurse, orthopaedic case manager, person conducting follow‐up telephone calls, stakeholders, colorectal surgical department	Implementation team: Chief nurse, unit director, project leader, nurses, nursing manager, orthopaedic case manager	Staff education about the intervention	5 (3)	(Hu et al. 2020; Lin et al. 2025, 2024; Mitchell 2022; Pelt et al. 2018)
Patient	Nurse	Patient education about the intervention (not as an intervention component)	4 (1)	(Robertson et al. 2018; Liu et al. 2019; Tian et al. 2023; Pelt et al. 2018)
**Implementation process strategies**				
Frontline, supervisory, leadership staff	Nurses, pharmacists, social workers, physicians, research team, nurse health education team	Monitor process/outcomes	9 (5)	(Hu et al. 2020; Lin et al. 2025; Tu et al. 2024; Du 2021; Fisher et al. 2018; Fitz et al. 2020; Mitchell 2022; Weintraub et al. 2018; Zuckerman 2020)
Not reported	Cardiac service team, project administrator, patients, nurses, surgeons, interdisciplinary lung transplant team, surgical staff	Assess context	6 (5)	(Du 2021; Fisher et al. 2018; Fitz et al. 2020; Koeckert et al. 2017; Pelt et al. 2018; Zuckerman 2020)
Not reported	Intervention team	Select change management approach	5 (1)	(Xu 2021; Du 2021; Fitz et al. 2020; Mitchell 2022; Zuckerman 2020)
Not reported	Physicians, inpatient and outpatient nursing staff, case managers, pharmacists, hospital administrators, clinical leaders, surgeons, frontline staff, supervisory staff, ‘providers’, patients, caregivers, nutritionists, psychological consultants, ostomy specialists, care administrators	Stakeholder engagement (in implementation process, not intervention development)	5 (5)	(Li et al. 2024; Zhou et al. 2023; Du 2021; Fisher et al. 2018; Pelt et al. 2018)
Not reported	Chief nurse	Determine project goals and measures of success	2 (1)	(Fisher et al. 2018; Mitchell 2022)
**Integration strategies**				
Associate chief nurse, attending physician, charge nurse, nurses, lead nurses, transitional care nurses, pharmacists, outpatient nurses and clinical staff, case managers, multi‐disciplinary team, kidney transplant surgeons, kidney transplant specialist nurses, medical assistant, project administrator, care navigator, nonclinical professional	Chief nurse, unit director, hospital administrators	Staffing changes and role clarification	11 (2)	(Li et al. 2020; Yang et al. 2023; Aicher et al. 2019; Du 2021; Fisher et al. 2018; Iseler et al. 2018; Mitchell 2022; Pelt et al. 2018; Zuckerman 2020; Coskun and Duygulu 2022; Ahmadi et al. 2021)
Follow‐up specialists, researcher	Not reported	Changes to electronic systems	7 (2)	(Li et al. 2020; Tian et al. 2023; Yang et al. 2023; Fisher et al. 2018; Fitz et al. 2020; Weintraub et al. 2018; Zuckerman 2020)
Nurse, caregivers	Researcher	Prompting	3 (1)	(Hu et al. 2020; Tseng et al. 2021; Grahn et al. 2019)
Not reported	Surgeon, nursing administrator, specific member of the surgery ward staff, e.g., dedicated wound/ostomy and surgical floor nurses	Champion	2 (2)	(Du 2021; Fisher et al. 2018)
Not reported	Hospital administrators	Financial resources	3 (1)	(Fisher et al. 2018; Pelt et al. 2018; Coskun and Duygulu 2022)
Catheterisation laboratory nursing and ancillary staff, Advanced practice provider	Not reported	Physical resources	2 (0)	(Aicher et al. 2019; Fisher et al. 2018)
Patients	Outpatient departments	Change routines and schedules	1 (1)	(Grahn et al. 2019)
Not reported	Not reported	Guidelines and policies	1 (0)	(Coskun and Duygulu 2022)
**Capacity‐building strategies**				
Not reported	Project leader, project administrator, medical coordinated‐transitional care originator, coordinated‐transitional care clinical leaders, champion	Project leader to direct implementation process	3 (3)	(Fisher et al. 2018; Mitchell 2022; Pelt et al. 2018)
Not reported	Program managers, chief physician, ostomy therapist/specialists, nurses, physician, care administrator	Ensure implementation staff adequately trained or possess the necessary expertise	3 (3)	(Zhang et al. 2020, 2021; Zhou et al. 2023; Weintraub et al. 2018)
**Scale‐up strategies**				
Patients	Not reported	State‐wide or nation‐wide program	2 (0)	(Zhou et al. 2023; Grahn et al. 2019)
Other institutions	Not reported	Prepare for dissemination to other institutions	1 (0)	(Fisher et al. 2018)

For integration strategies, the most frequently used strategies were changes to staffing such as hiring staff, role clarification (Du 2021; Fisher et al. 2018; Ahmadi et al. 2021), re‐allocating staff or some of their time (Iseler et al. 2018; Coskun and Duygulu 2022), and integrating new and existing roles (Fisher et al. 2018). Only two studies reported people responsible, which included senior staff such as chief nurses, unit directors and hospital administrations. The second most common integration strategy was changes to electronic systems, including implementing electronic tools for quality monitoring (Zuckerman 2020), adding workflows (Fisher et al. 2018), platforms (Yang et al. 2023; Weintraub et al. 2018) and databases (Li et al. 2020; Tian et al. 2023), and integrating forms into the electronic medical record (Fitz et al. 2020); people responsible were not reported.

Monitoring processes and outcomes, assessing the context, and selecting change management approaches were common implementation process strategies. Examples of monitoring processes and outcomes included readmission data being regularly reviewed for lessons learnt (Weintraub et al. 2018), metrics reported to frontline staff (Du 2021), and in one study intervention fidelity was checked weekly with a WeChat group created to share issues with the implementation process and ensure these were promptly addressed (Hu et al. 2020). Assessing the context tended to focus on examining existing processes and issues in the hospitals (Du 2021; Fisher et al. 2018; Fitz et al. 2020; Koeckert et al. 2017; Pelt et al. 2018; Zuckerman 2020), while stakeholder engagement incorporated a range of hospital staff, as well as patients and caregivers to support the implementatin process (Lin et al. 2024; Zhou et al. 2023; Du 2021; Fisher et al. 2018; Pelt et al. 2018). Finally, change management approaches such as ‘Plan, Do, Study, Act’ (Fitz et al. 2020; Mitchell 2022; Zuckerman 2020), and ‘Promoting Action on Research Implementation in Health Services’ (PARIHS) framework (Xu 2021) were used. People responsible for implementation process strategies were both frontline clinicians, as well as project administrators and research teams members. Table 2 details the synthesis of findings using the PAGER Framework.

**TABLE 2:** Patterns, advances, gaps, evidence for practice and research recommendations framework.

Patterns	Advances	Gaps	Evidence for practice	Research recommendations
Implementation strategies for surgical TCIs are targeted at the delivery system (i.e., those individuals, teams and systems that must adopt the intervention into practice).	There is growing evidence that implementation strategies needed to support surgical TCI implementation include: – Providing adequate staffing. – Developing or changing electronic systems. – Educating multi‐disciplinary clinicians to deliver the intervention. – Using change management processes.	Few implementation strategies were targeted at the support system (i.e., those individuals, teams and systems that build capacity to adopt the intervention): – In a few instances a project leader was hired to manage the change process; their skills/training was rarely reported. – Few interventions were scaled up; often surgical TCIs are context specific, approaches to transferring TCIs to other settings was unclear in this review. – Although two studies reported increased financial resources provided for surgical TCIs, the financial viability of strategies such as staffing and electronic system changes is unclear. – Implementation fidelity and intervention fidelity were underreported.	– Hospital leader support is critical to implementing surgical TCIs; they must provide resources such as staffing, including up‐skilled staff to manage the change management process and have adequate IT departments to make changes to electronic systems. – Staff member/s responsible for implementing the surgical TCI could use strategies outlined in this review. For example, they could provide specific education to multi‐disciplinary teams and use change management processes.	– Consider using strategies outlined in this review when implementing and evaluating surgical TCIs. – Report intervention fidelity as well as implementation fidelity for all implementation strategies used. – Develop and test scale‐up strategies in the context of implementation trials, to demonstrate how to best adapt surgical TCIs across settings. – Some implementation strategies appear costly; economic analyses to demonstrate the benefits of increased costs on outcomes like hospital readmission could be beneficial. – Consider reporting the qualifications/training of the person responsible for implementing the surgical TCI to make reporting transparent and replication easier.

## 5 Discussion

In this scoping review, the most common implementation strategies for surgical TCIs were dissemination strategies (i.e., staff education and training), integration strategies (i.e., staff changes and changes to electronic systems), and implementation process strategies (i.e., change management approaches including assessing the context and monitoring). As per Leeman et al. (2017), all of these implementation strategies are targeted at individuals, teams and systems that must use the intervention in practice (i.e., the delivery system). Less common strategies were capacity‐building strategies for those responsible for leading the change initiative and scale‐up strategies. These strategies help individuals, teams and systems build capacity to adopt the intervention (i.e., the support system) (Leeman et al. 2017). The implementation strategies we found were largely used in the US and Asian countries with patients undergoing colorectal and complex abdominal surgery or cardiac surgery; these contextual factors must be considered by those intending to use the strategies in their setting.

Building staff capacity was brought to the fore in this scoping review. Staff education was a common implementation strategy, which contrasts with previous TCI research that showed detailed training is often lacking, creating a barrier to successful implementation (Fakha et al. 2021; Sadler et al. 2019). As found in our scoping review, the target audience requiring education can be extensive depending on whether a monocentric (i.e., one professional coordinates the whole TCI process) or polycentric (i.e., responsibility for the TCI process is shared amongst a multi‐disciplinary team) approach to surgical TCIs is undertaken (Landi et al. 2024). Yet, capacity‐building for the person responsible for driving the change management process was infrequent in our scoping review. Previous research has shown that those responsible for managing change processes in hospitals often have no formal training in change management, they learn through mentors who may promote misinformation, and they misapply change management approaches like PDSA cycles (Wright et al. 2022). Given that implementation process strategies, such as using change management approaches, were frequent in our scoping review, it underlines the importance of providing training for those leading the change management process, in addition to those delivering the intervention (Lewis and Kulhanek 2022).

Our scoping review highlights the importance of harmonising the resources needed to implement surgical TCIs with desired outcomes like cost‐savings. It was evident in our scoping review that financial support from hospital leaders was required for high‐cost integration strategies like staffing and changes to electronic systems; strategies found to facilitate TCI implementation in other reviews (Fakha et al. 2021; Sadler et al. 2019). Lack of staffing is a common reason TCI implementation fails (Fakha et al. 2021; Sadler et al. 2019), especially if staff in existing roles are expected to take on additional tasks to facilitate the TCI (Fakha et al. 2023). Creating a new role for a designated person to coordinate the whole TCI, often a master's prepared nurse, is a common approach that provides continuity of care for patients and their caregivers, but adds to hospital budgets (Fakha et al. 2021, 2023; Sadler et al. 2019). Overall, further research is needed to demonstrate the cost benefits of surgical TCIs, as previous research has only provided partial economic evaluations, that were descriptive in manner or had issues with quality (Landi et al. 2024; Kast et al. 2021). In the current climate where hospitals are financially constrained and workload and staffing issues are rife (Fakha et al. 2021, 2023); there is a real need to show that implementation strategies result in outcomes, to convince hospital leaders of the need for continuous funding for this approach to care.

Scale‐up strategies were rarely trialled for the surgical TCIs included in this scoping review. TCIs are complex interventions (Gesell et al. 2021) that must be adapted to the population and environment, and coupled with context‐specific implementation strategies, to reduce implementation failure when scaled up (Fakha et al. 2023). Considering that studies included in our scoping review were largely conducted with patients undergoing colorectal and cardiac surgery in Asian countries and the US, future adaptation of these interventions is likely needed to ensure a match between the intervention and the population/setting. A suggested approach is to preserve function (i.e., the core purpose of an intervention or intervention component), but change form (i.e., activities used to carry out functions of intervention, such as intended mode of delivery, materials, intervention dose or providers involved) when transferring surgical TCIs across settings; as form often requires adaptation to make interventions acceptable for the population and setting (Fann et al. 2021). Currently, many TCI researchers define intervention form and measure intervention fidelity, rather than function (Gesell et al. 2021). An increased focus on TCI function would facilitate standardisation of TCI interventions across settings and support maintenance of intervention effectiveness (Gesell et al. 2021; Hawe 2015). However, agreement on the function of surgical TCIs is needed, presenting a future research opportunity. Recently, researchers identified that ideal TCI components for achieving outcomes like reduced readmission included monitoring and managing symptoms after discharge and discharge planning, which must be combined with either staff coordination or patient education and social and community support (Landi et al. 2024). To further enhance the success of scale‐up, context‐specific and theory‐informed implementation strategies ought to be selected and implementation fidelity should be measured and reported (Proctor et al. 2013). Without this, it will be difficult to interpret why the intervention worked or not (Proctor et al. 2013). In summary, knowing the essential function of surgical TCIs would reduce the need for reinventing solutions and selecting overly intensive intervention components, while facilitating the comparison of data across settings for researchers; however, implementation considerations are just as important for assessing the true effectiveness of the intervention (Hawe 2015).

### 5.1 Recommendations for Clinical Practice and Further Research

We recommend high‐quality change management processes, led by qualified people, be used when implementing TCIs. This could include careful assessment of the context to determine implementation strategies required. Importantly, these strategies should be theory‐informed as this approach is more likely to ensure behaviour change and successful implementation. For future research, we suggest investigation of surgical TCI adaptation, in a way that maintains their function. This could be coupled with testing of the implementation strategies found in this scoping review and measurement of implementation fidelity. Finally, economic evaluations of surgical TCIs are necessary to provide compelling data to increase hospital leader buy‐in for surgical TCI implementation.

### 5.2 Strengths and Limitations

In terms of study strengths, first, we used a systematic approach when conducting the search and screening, however, there is always the chance that eligible studies were missed. Second, we included both research and quality improvement to provide a more holistic view of the surgical TCI landscape; QI can be viewed as lower quality than research but may provide practical implementation strategies that work in the real world. For limitations, first, we intended to use the Expert Recommendations for Implementing Change (ERIC) implementation strategies framework to identify patterns, as per our published protocol. However, there were 73 ERIC implementation strategies making coding challenging, and as Powell et al. (2015) concluded, ERIC implementation strategies require ongoing work to make them into conceptually distinct categories. We believe that using Leeman et al.'s (2017) classes instead provided a clear and organised framework that could be understood by both clinicians and researchers, aiding practical implementation efforts. Further, scoping review methodology is flexible, allowing adaptations such as this. Finally, the results are limited as there was homogeneity in context and surgical populations; thus, readers must consider the generalisability of the implementation strategies for their context.

## 6 Conclusions

Our scoping review provides a synthesis of previously used implementation strategies for surgical TCIs, which often include staff education, adequate staffing, changes to electronic systems, and change management processes. Many of these strategies will likely result in increased financial resources, highlighting the need for hospital leader support and evidence of the financial sustainability of surgical TCIs. Underexplored implementation strategies included evaluation of how to scale up TCIs considering their context‐specific nature and capacity‐building to ensure high‐quality change management processes. On the whole, reporting of implementation and intervention fidelity is lacking, and research has been largely confined to Asia and the US with patients who experience colorectal, complex abdominal and cardiac surgery. Overall, there is much work to be done to ensure surgical TCIs are implemented in the real world effectively, to achieve their intended benefits.

## Author Contributions

G.T.: conceptualisation, data curation, formal analysis, investigation, methodology, project administration, validation, visualisation, writing – original draft, writing – review and editing. B.M.G.: conceptualisation, formal analysis, methodology, resources, validation, visualisation, supervision, writing – review and editing. K.T.: data curation, formal analysis, investigation, methodology, project administration, validation, visualisation, writing – review and editing. A.M.E.: formal analysis, investigation, validation, visualisation, writing – review and editing. B.P.: formal analysis, investigation, validation, visualisation, writing – review and editing. J.C.: formal analysis, validation, visualisation, writing – review and editing. L.F.: formal analysis, validation, visualisation, writing – original draft, writing – review and editing. W.C.: conceptualisation, formal analysis, investigation, methodology, resources, validation, visualisation, supervision, writing – review and editing.

## Ethics Statement

The authors have nothing to report.

## Consent

The authors have nothing to report.

## Conflicts of Interest

The authors declare no conflicts of interest.

## Supporting information


**File S1.** Search strategy in Medline (EBSCOhost).

**File S2.** Patient partner engagement in scoping review, informed by GRIPP2‐SF reporting guidelines.

**File S3.** Study characteristics (*N* = 27 studies).

## Data Availability

Data analysed in the current research were a re‐investigation of existing data, which are openly available in the computerised databases named in this publication.

## References

[jan70081-bib-0001] Ahmadi, N. , L. Mbuagbaw , C. Finley , J. Agzarian , W. C. Hanna , and Y. Shargall . 2021. “Impact of the Integrated Comprehensive Care Program Post‐Thoracic Surgery: A Propensity Score–Matched Study.” Journal of Thoracic and Cardiovascular Surgery 162, no. 1: 321–330. 32713635 10.1016/j.jtcvs.2020.05.095

[jan70081-bib-0002] Aicher, B. O. , E. Hanlon , S. Rosenberger , S. Toursavadkohi , and R. S. Crawford . 2019. “Reduced Length of Stay and 30‐Day Readmission Rate on an Inpatient Vascular Surgery Service.” Journal of Vascular Nursing 37, no. 2: 78–85. 31155166 10.1016/j.jvn.2018.11.004 PMC6548444

[jan70081-bib-0003] Arksey, H. , and L. O'Malley . 2005. “Scoping Studies: Towards a Methodological Framework.” International Journal of Social Research Methodology 8, no. 1: 19–32.

[jan70081-bib-0004] Bauer, M. S. , and J. Kirchner . 2020. “Implementation Science: What Is It and Why Should I Care?” Psychiatry Research 283: 112376. 31036287 10.1016/j.psychres.2019.04.025

[jan70081-bib-0005] Bradbury‐Jones, C. , H. Aveyard , O. R. Herber , L. Isham , J. Taylor , and L. O’Malley . 2021. “Scoping Reviews: The PAGER Framework for Improving the Quality of Reporting.” International Journal of Social Research Methodology 25, no. 4: 457–470. 10.1080/13645579.2021.1899596.

[jan70081-bib-0006] Brown, C. S. , J. R. Montgomery , P. U. Neiman , et al. 2021. “Assessment of Potentially Preventable Hospital Readmissions After Major Surgery and Association With Public vs Private Health Insurance and Comorbidities.” JAMA Network Open 4, no. 4: e215503. 33847752 10.1001/jamanetworkopen.2021.5503 PMC8044735

[jan70081-bib-0007] Coskun, S. , and S. Duygulu . 2022. “The Effects of Nurse Led Transitional Care Model on Elderly Patients Undergoing Open Heart Surgery: A Randomized Controlled Trial.” European Journal of Cardiovascular Nursing 21, no. 1: 46–55. 33821999 10.1093/eurjcn/zvab005

[jan70081-bib-0008] Craig, P. , P. Dieppe , S. Macintyre , S. Michie , I. Nazareth , and M. Petticrew . 2008. “Developing and Evaluating Complex Interventions: The New Medical Research Council Guidance.” BMJ 337: a1655. 18824488 10.1136/bmj.a1655 PMC2769032

[jan70081-bib-0009] Du, R. Y. 2021. “Implementation and Feasibility of the Re‐Engineered Discharge for Surgery (RED‐S) Intervention: A Pilot Study.” Journal for Healthcare Quality: Official Publication of the National Association for Healthcare Quality 43, no. 2: 92–100. 32544139 10.1097/JHQ.0000000000000266 PMC9825132

[jan70081-bib-0010] Eskes, A. M. , G. Tobiano , J. Carlini , C. Kuijpers , S. C. W. Musters , and W. Chaboyer . 2023. “Fundamentally Shifting Discharge Planning and Post‐Hospital Care.” International Journal of Nursing Studies 145: 104533. 37285731 10.1016/j.ijnurstu.2023.104533

[jan70081-bib-0011] Fakha, A. , L. Groenvynck , B. de Boer , T. van Achterberg , J. Hamers , and H. Verbeek . 2021. “A Myriad of Factors Influencing the Implementation of Transitional Care Innovations: A Scoping Review.” Implementation Science 16, no. 1: 21. 33637097 10.1186/s13012-021-01087-2 PMC7912549

[jan70081-bib-0012] Fakha, A. , M. Leithaus , B. de Boer , T. van Achterberg , J. P. Hamers , and H. Verbeek . 2023. “Implementing Four Transitional Care Interventions for Older Adults: A Retrospective Collective Case Study.” Gerontologist 63, no. 3: 451–466. 36001088 10.1093/geront/gnac128 PMC10028228

[jan70081-bib-0013] Fann, J. R. , T. Hart , M. A. Ciol , et al. 2021. “Improving Transition From Inpatient Rehabilitation Following Traumatic Brain Injury: Protocol for the BRITE Pragmatic Comparative Effectiveness Trial.” Contemporary Clinical Trials 104: 106332. 33652127 10.1016/j.cct.2021.106332

[jan70081-bib-0014] Fiona, P. 2014. “Effectiveness and Implementation of Enhanced Recovery After Surgery Programmes: A Rapid Evidence Synthesis.” BMJ Open 4, no. 7: e005015. 10.1136/bmjopen-2014-005015 PMC4120402 25052168

[jan70081-bib-0015] Fisher, A. V. , S. A. Campbell‐Flohr , L. Sell , E. Osterhaus , A. W. Acher , and K. Leahy‐Gross . 2018. “Adaptation and Implementation of a Transitional Care Protocol for Patients Undergoing Complex Abdominal Surgery.” Joint Commission Journal on Quality and Patient Safety 44, no. 12: 741–750. 30097384 10.1016/j.jcjq.2018.05.001 PMC7474978

[jan70081-bib-0016] Fitz, S. , L. Diegel‐Vacek , and E. Mahoney . 2020. “A Performance Improvement Initiative for Implementing an Evidence‐Based Discharge Bundle for Lung Transplant Recipients.” Progress in Transplantation 30, no. 3: 281–285. 32552376 10.1177/1526924820933832

[jan70081-bib-0017] Gesell, S. B. , J. Prvu Bettger , R. H. Lawrence , et al. 2021. “Implementation of Complex Interventions: Lessons Learned From the Patient‐Centered Outcomes Research Institute Transitional Care Portfolio.” Medical Care 59, no. 4: S344–s354. 34228016 10.1097/MLR.0000000000001591 PMC8263141

[jan70081-bib-0018] Grahn, S. W. , A. C. Lowry , M. C. Osborne , et al. 2019. “System‐Wide Improvement for Transitions After Ileostomy Surgery: Can Intensive Monitoring of Protocol Compliance Decrease Readmissions? A Randomized Trial.” Diseases of the Colon & Rectum 62, no. 3: 363–370. 30489324 10.1097/DCR.0000000000001286

[jan70081-bib-0019] Hawe, P. 2015. “Lessons From Complex Interventions to Improve Health.” Annual Review of Public Health 36, no. 1: 307–323. 10.1146/annurev-publhealth-031912-114421 25581153

[jan70081-bib-0020] Hirschman, K. B. , K. Hirschman , E. Shaid , K. McCauley , M. Pauly , and M. Naylor . 2015. “Continuity of Care: The Transitional Care Model.” Online Journal of Issues in Nursing 20, no. 3: 1. 26882510

[jan70081-bib-0021] Holland, D. E. , and M. R. Harris . 2007. “Discharge Planning, Transitional Care, Coordination of Care, and Continuity of Care: Clarifying Concepts and Terms From the Hospital Perspective.” Home Health Care Services Quarterly 26, no. 4: 3–19. 18032197 10.1300/J027v26n04_02

[jan70081-bib-0022] Hu, R. , B. Gu , Q. Tan , et al. 2020. “The Effects of a Transitional Care Program on Discharge Readiness, Transitional Care Quality, Health Services Utilization and Satisfaction Among Chinese Kidney Transplant Recipients: A Randomized Controlled Trial.” International Journal of Nursing Studies 110: 103700. 32739670 10.1016/j.ijnurstu.2020.103700

[jan70081-bib-0023] Iseler, J. , J. Fox , and K. Wierenga . 2018. “Performance Improvement to Decrease Readmission Rates for Patients With a Left Ventricular Assist Device.” Progress in Transplantation 28, no. 2: 184–188. 29558876 10.1177/1526924818765820 PMC5999042

[jan70081-bib-0024] Jones, C. E. , R. H. Hollis , T. S. Wahl , et al. 2016. “Transitional Care Interventions and Hospital Readmissions in Surgical Populations: A Systematic Review.” American Journal of Surgery 212, no. 2: 327–335. 27353404 10.1016/j.amjsurg.2016.04.004

[jan70081-bib-0025] Kang, E. , B. M. Gillespie , G. Tobiano , and W. Chaboyer . 2020. “General Surgical Patients' Experience of Hospital Discharge Education: A Qualitative Study.” Journal of Clinical Nursing 29, no. 1: e1–e10. 31509311 10.1111/jocn.15057

[jan70081-bib-0026] Kast, K. , C. P. Wachter , O. Schöffski , and M. Rimmele . 2021. “Economic Evidence With Respect to Cost‐Effectiveness of the Transitional Care Model Among Geriatric Patients Discharged From Hospital to Home: A Systematic Review.” Europena Journal of Health Economics 22, no. 6: 961–975. 10.1007/s10198-021-01301-4 PMC8275561 33839965

[jan70081-bib-0027] Koeckert, M. S. , P. A. Ursomanno , M. R. Williams , et al. 2017. “Reengineering Valve Patients' Postdischarge Management for Adapting to Bundled Payment Models.” Journal of Thoracic and Cardiovascular Surgery 154, no. 1: 190–198. 28412109 10.1016/j.jtcvs.2016.10.109

[jan70081-bib-0028] Landi, S. , M. M. Panella , and C. Leardini . 2024. “Disentangling Organizational Levers and Economic Benefits in Transitional Care Programs: A Systematic Review and Configurational Analysis.” BMC Health Services Research 24, no. 1: 46. 38195545 10.1186/s12913-023-10461-3 PMC10777542

[jan70081-bib-0029] Leeman, J. , S. A. Birken , B. J. Powell , C. Rohweder , and C. M. Shea . 2017. “Beyond ‘Implementation Strategies’: Classifying the Full Range of Strategies Used in Implementation Science and Practice.” Implementation Science 12, no. 1: 125. 29100551 10.1186/s13012-017-0657-x PMC5670723

[jan70081-bib-0030] Lewis, D. , and B. Kulhanek . 2022. “Managing Change Through Training.” In Healthcare Technology Training: An Evidence‐Based Guide for Improved Quality, edited by B. Kulhanek and K. Mandato , 33–55. Springer International Publishing.

[jan70081-bib-0031] Li, L. , Z. Ma , and W. Wang . 2020. “Influence of Transitional Care on the Self‐Care Ability of Kidney Transplant Recipients After Discharge.” Annals of Palliative Medicine 9, no. 4: 1951–1958. 10.21037/apm-20-1120 32692223

[jan70081-bib-0032] Li, M. , K. Yu , Y. J. Zhang , A. Mao , and L. Y. Dong . 2024. “Impact of Discharge Planning Combined With ‘Internet Home Ostomy Care Platform’ in Patients With Permanent Colostomy After Rectal Cancer Surgery.” Annali Italiani di Chirurgia 95, no. 4: 699–707. 39186342 10.62713/aic.3459

[jan70081-bib-0033] Lin, L. , Y. Fang , Y. Wei , F. Huang , J. Zheng , and H. Xiao . 2024. “The Effects of a Nurse‐Led Discharge Planning on the Health Outcomes of Colorectal Cancer Patients With Stomas: A Randomized Controlled Trial.” International Journal of Nursing Studies 155: 104769. 38676992 10.1016/j.ijnurstu.2024.104769

[jan70081-bib-0034] Lin, L. , N. Lin , M. Yan , J. Xie , R. Lin , and H. Lin . 2025. “The Effect of a Nurse‐Led Health Education Model for Patients With Temporary Stomas: A Randomized Controlled Trial.” European Journal of Oncology Nursing 76: 102861. 40117906 10.1016/j.ejon.2025.102861

[jan70081-bib-0035] Liu, J. , N. Gormley , H. H. Dasenbrock , et al. 2019. “Cost‐Benefit Analysis of Transitional Care in Neurosurgery.” Neurosurgery 85, no. 5: 672–679. 30272201 10.1093/neuros/nyy424

[jan70081-bib-0036] Mao, H. , Y. Xie , Y. Shen , M. Wang , and Y. Luo . 2022. “Effectiveness of Nurse‐Led Discharge Service on Adult Surgical Inpatients: A Meta‐Analysis of Randomized Controlled Trials.” Nursing Open 9, no. 5: 2250–2262. 35661429 10.1002/nop2.1268 PMC9374412

[jan70081-bib-0037] Mitchell, K. 2022. “Impact of Reengineered Discharge Toolkit on Patients Undergoing Total Joint Surgeries.” Rehabilitation Nursing 47, no. 4: 121–128. 35701987 10.1097/RNJ.0000000000000375

[jan70081-bib-0038] Morris, M. S. , R. J. Deierhoi , J. S. Richman , L. K. Altom , and M. T. Hawn . 2014. “The Relationship Between Timing of Surgical Complications and Hospital Readmission.” JAMA Surgery 149, no. 4: 348–354. 24522747 10.1001/jamasurg.2013.4064

[jan70081-bib-0039] Naylor, M. D. , L. H. Aiken , E. T. Kurtzman , D. M. Olds , and K. B. Hirschman . 2011. “The Care Span: The Importance of Transitional Care in Achieving Health Reform.” Health Affairs 30, no. 4: 746–754. 21471497 10.1377/hlthaff.2011.0041

[jan70081-bib-0040] Pelt, C. E. , J. M. Gililland , J. A. Erickson , D. E. Trimble , M. B. Anderson , and C. L. Peters . 2018. “Improving Value in Total Joint Arthroplasty: A Comprehensive Patient Education and Management Program Decreases Discharge to Post‐Acute Care Facilities and Post‐Operative Complications.” Journal of Arthroplasty 33, no. 1: 14–18. 28887021 10.1016/j.arth.2017.08.003

[jan70081-bib-0041] Peters, M. 2015. “The Joanna Briggs Institute Reviewers' Manual 2015: Methodology for JBI Scoping Reviews.”

[jan70081-bib-0042] Powell, B. J. , T. J. Waltz , M. J. Chinman , et al. 2015. “A Refined Compilation of Implementation Strategies: Results From the Expert Recommendations for Implementing Change (ERIC) Project.” Implementation Science 10, no. 1: 21. 25889199 10.1186/s13012-015-0209-1 PMC4328074

[jan70081-bib-0043] Proctor, E. K. , B. J. Powell , and J. C. McMillen . 2013. “Implementation Strategies: Recommendations for Specifying and Reporting.” Implementation Science 8, no. 1: 139. 24289295 10.1186/1748-5908-8-139 PMC3882890

[jan70081-bib-0044] Rennke, S. , O. K. Nguyen , M. H. Shoeb , Y. Magan , R. M. Wachter , and S. R. Ranji . 2013. “Hospital‐Initiated Transitional Care Interventions as a Patient Safety Strategy.” Annals of Internal Medicine 158, no. 5: 433–440. 23460101 10.7326/0003-4819-158-5-201303051-00011

[jan70081-bib-0045] Robertson, F. C. , J. L. Logsdon , H. H. Dasenbrock , et al. 2018. “Transitional Care Services: A Quality and Safety Process Improvement Program in Neurosurgery.” Journal of Neurosurgery 128, no. 5: 1570–1577. 28707992 10.3171/2017.2.JNS161770

[jan70081-bib-0046] Sadler, E. , V. Potterton , R. Anderson , et al. 2019. “Service User, Carer and Provider Perspectives on Integrated Care for Older People With Frailty, and Factors Perceived to Facilitate and Hinder Implementation: A Systematic Review and Narrative Synthesis.” PLoS One 14, no. 5: e0216488. 31083707 10.1371/journal.pone.0216488 PMC6513075

[jan70081-bib-0047] Staniszewska, S. , J. Brett , I. Simera , et al. 2017. “GRIPP2 Reporting Checklists: Tools to Improve Reporting of Patient and Public Involvement in Research.” BMJ 358: j3453. 28768629 10.1136/bmj.j3453 PMC5539518

[jan70081-bib-0048] Tian, T. , M. J. Guan , L. J. Liu , X. Q. Su , H. Wang , and L. He . 2023. “Study on the Efficacy of ‘Information Platform+ Self‐Care Model’ on the Health Status of Discharged Patients Following Vaginal Natural Orifice Transluminal Endoscopic Surgery.” International Journal of Women's Health 15: 1185–1195. 10.2147/IJWH.S416134 PMC10386867 37520183

[jan70081-bib-0049] Tobiano, G. , W. Chaboyer , K. Turner , et al. 2025. “Surgical Transitional Care Interventions and Their Outcomes: A Scoping Review.” International Journal of Nursing Studies Advances 8, no. 8: 100328. 40469576 10.1016/j.ijnsa.2025.100328 PMC12136900

[jan70081-bib-0050] Tricco, A. C. , E. Lillie , W. Zarin , et al. 2018. “PRISMA Extension for Scoping Reviews (PRISMA‐ScR): Checklist and Explanation.” Annals of Internal Medicine 169, no. 7: 467–473. 30178033 10.7326/M18-0850

[jan70081-bib-0051] Tseng, M.‐Y. , C. T. Yang , J. Liang , et al. 2021. “A Family Care Model for Older Persons With Hip‐Fracture and Cognitive Impairment: A Randomized Controlled Trial.” International Journal of Nursing Studies 120: 103995. 34146844 10.1016/j.ijnurstu.2021.103995

[jan70081-bib-0052] Tu, J. , J. Zhou , X. Li , Q. Zhang , M. Luo , and J. Zhou . 2024. “Effectiveness of the 5As Model‐Based Transitional Care Program Among Chinese Patients With Type B Aortic Dissection Post‐TEVAR: A Randomized Controlled Trial.” Reviews in Cardiovascular Medicine 25, no. 9: 347. 39355579 10.31083/j.rcm2509347 PMC11440392

[jan70081-bib-0053] Wang, S. , and F. Gao . 2025. “Using Orem's Self‐Care Model for a Continuing Care Program After Transurethral Prostate Resection.” Research and Theory for Nursing Practice, Springer. 10.1891/RTNP-2024-0159 40121010

[jan70081-bib-0054] Weintraub, W. S. , D. Elliott , Z. Fanari , et al. 2018. “The Impact of Care Management Information Technology Model on Quality of Care After Coronary Artery Bypass Surgery: ‘Bridging the Divides’.” Cardiovascular Revascularization Medicine 19, no. 1: 106–111. 28651834 10.1016/j.carrev.2017.06.008 PMC5740011

[jan70081-bib-0055] World Health Organization . 2016. “Transitions of Care.” https://apps.who.int/iris/handle/10665/252272.

[jan70081-bib-0056] Wright, D. , J. Gabbay , and A. Le May . 2022. “Determining the Skills Needed by Frontline NHS Staff to Deliver Quality Improvement: Findings From Six Case Studies.” BMJ Quality and Safety 31, no. 6: 450–461. 10.1136/bmjqs-2021-013065 PMC9132850 34452950

[jan70081-bib-0057] Xu, Y.‐p. 2021. “The Effect of Care Transition Pathway Implementation on Patients Undergoing Joint Replacement During the COVID‐19 Pandemic: A Quasi‐Experimental Study From a Tertiary Care Hospital Orthopedic Department in Beijing, China.” Journal of Orthopaedic Surgery and Research 16, no. 1: 1–5. 34074300 10.1186/s13018-021-02511-5 PMC8167389

[jan70081-bib-0058] Yang, W. , H. Xu , W. Miao , Z. Geng , and G. Geng . 2023. “Effects of Transitional Care Based on the Social Support Theory for Older Patients With Osteoporotic Vertebral Compression Fractures: A Quasi‐Experimental Trial.” Australasian Journal on Ageing 42, no. 1: 185–194. 35996354 10.1111/ajag.13129

[jan70081-bib-0059] Zhang, X. , R. Gao , J. L. Lin , et al. 2020. “Effects of Hospital‐Family Holistic Care Model on the Health Outcome of Patients With Permanent Enterostomy Based on the Theory of ‘Timing It Right’.” Journal of Clinical Nursing 29, no. 13: 2196–2208. 31970830 10.1111/jocn.15199

[jan70081-bib-0060] Zhang, X. , J. L. Lin , R. Gao , et al. 2021. “Application of the Hospital‐Family Holistic Care Model in Caregivers of Patients With Permanent Enterostomy: A Randomized Controlled Trial.” Journal of Advanced Nursing 77, no. 4: 2033–2049. 33523488 10.1111/jan.14691

[jan70081-bib-0061] Zhou, L. , F. Zhang , H. Li , and L. Wang . 2023. “Post‐Discharge Health Education for Patients With Enterostomy: A Nationwide Interventional Study.” Journal of Global Health 13: 4172. 10.7189/jogh.13.04172 PMC10716631 38085224

[jan70081-bib-0062] Zuckerman, S. L. 2020. “The Institute for Healthcare Improvement–NeuroPoint Alliance Collaboration to Decrease Length of Stay and Readmission After Lumbar Spine Fusion: Using National Registries to Design Quality Improvement Protocols.” Journal of Neurosurgery: Spine 33, no. 6: 812–821. 32823267 10.3171/2020.5.SPINE20457

