# Supplementary material for: Implementing Transitional Care Interventions for Surgical Patients: A Scoping Review
Source: J Adv Nurs. 2025 Jul 21;82(4):2617–29. doi: 10.1111/jan.70081 (PMC12994648; doi:10.1111/jan.70081)
Supplement: Supplementary file 1 — File S1. Search strategy in Medline (EBSCOhost). [file JAN-82-2617-s003.docx]

**Supplementary File 1.**

Search strategy in Medline (EBSCOhost)

| **#** | **Query** |
| --- | --- |
| S9 | Limiters - Date of Publication  Limiters - Age |
| S8 | S3 AND S7 |
| S7 | S4 OR S5 OR S6 |
| S6 | (MH "Transitional Care") |
| S5 | ((TI Transition* OR AB Transition*) N2 (TI care OR AB care)) |
| S4 | (((TI Transition* OR AB Transition*) OR (TI discharge OR AB discharge) OR (TI postdischarge OR AB postdischarge) OR (TI post-discharge OR AB post-discharge)) N2 ((TI intervention OR AB intervention) OR (TI program OR AB program) OR (TI service OR AB service) OR (TI pathway OR AB pathway) OR (TI plan* OR AB plan*))) |
| S3 | S1 OR S2 |
| S2 | AB ( perioperative or peri-operative or pre-operative or preoperative or post-operative or postoperative or surg* or operat* ) OR TI ( perioperative or peri-operative or pre-operative or preoperative or post-operative or postoperative or surg* or operat* ) |
| S1 | (MH "Surgical Procedures, Operative+") |
