# Supplementary material for: Implementing Transitional Care Interventions for Surgical Patients: A Scoping Review
Source: J Adv Nurs. 2025 Jul 21;82(4):2617–29. doi: 10.1111/jan.70081 (PMC12994648; doi:10.1111/jan.70081)
Supplement: Supplementary file 2 — File S2. Patient partner engagement in scoping review, informed by GRIPP2‐SF reporting guidelines. [file JAN-82-2617-s001.docx]

**Supplementary File 2.** Patient partner engagement in scoping review, informed by GRIPP2-SF reporting guidelines [1]

| Section and topic | Item |
| --- | --- |
| 1: Aim  Report the aim of patient and public engagement (PPE) in the study | The aim was to incorporate health consumer perspectives into the scoping review, to provide alternative viewpoints to academic researchers. |
| 2: Methods  Provide a clear description of the methods used for PPE in the study | Prior to commencing the review our patient partner Janelle had a meeting with the lead research to share her lived experiences related to being discharged from hospital after surgery.  Janelle interpreted the findings of the review to inform the discussion. |
| 3: Results  Outcomes: Report the results of PPE in the study, including both positive and negative outcomes | • Focus of review: Janelle agreed that transitional care after surgery was an important area for research investigation, as there were many issues/gaps in care identified by Janelle.  • Discussion: Janelle reviewed the findings and provided interpretations of the meaning of the findings, based on how they aligned or did not align with her experiences. |
| 4: Discussion  Outcomes: Comment on the extent to which PPE influenced the study overall. Describe positive and negative effects | • Focus of review: Research questions that were important to patients were investigated.  • Discussion: Janelle’s interpretations were developed into main discussion ideas, which were used to write the discussion. Janelle checked the final version of the discussion and to check her interpretations were captured correctly. |
| 5: Reflections  Critical perspective: Comment critically on the study, reflecting on the things that went well and those that did not, so others can learn from this experience | • From Janelle’s perspective, it was critical to have discussions early in the process of the scoping review, not when the review was too far progressed. Confirming the importance of the review topic prior to starting was something that was done well.  • From the researchers’ perspectives Janelle’s interpretations provided an additional perspective and gave the research team inspiration when writing the discussion. |

1. Staniszewska, S., et al., *GRIPP2 reporting checklists: tools to improve reporting of patient and public involvement in research.* BMJ, 2017. **358**: p. j3453.
