# Supplementary material for: Implementing Transitional Care Interventions for Surgical Patients: A Scoping Review
Source: J Adv Nurs. 2025 Jul 21;82(4):2617–29. doi: 10.1111/jan.70081 (PMC12994648; doi:10.1111/jan.70081)
Supplement: Supplementary file 3 — File S3. Study characteristics (N = 27 studies). [file JAN-82-2617-s002.docx]

**Supplementary File 3.** Study characteristics (N=27 studies)

| Author, year, country | **Setting and surgery type** | Implementation fidelity | Intervention fidelity |
| --- | --- | --- | --- |
| Ahmadi et al., 2021; Canada [54] | **Setting:** Tertiary care academic institution  **Surgery type:** Lung resection | Not reported | Not reported |
| Aicher et al., 2019; USA [42] | **Setting:** Medical-surgical telemetry units of university medical centre  **Surgery type:** Vascular surgery including aortic surgery, ruptured aortic aneurysm, lower extremity amputation, lower extremity open surgery | Physical resources: Clinical space was repurposed  Other implementation strategies: Not reported | Not reported |
| Coskun & Duygulu, 2022; Turkey [53] | **Setting:** One cardiovascular surgery clinic and a university hospital  **Surgery type**: Open heart | Not reported | Not reported |
| Du et al., 2021; USA [43] | **Setting:** Veterans Affairs tertiary hospital  **Surgery type:** Colectomy | Staffing changes and role clarification: Delays in hiring new staff  Select change management approach: Approach was selected  Other implementation strategies: Not reported | - Postoperative education on wound care completed: n=21/21 (100%)  - Enterostomy education for patients with ostomy completed: n=7/9 (77%)  - Surgery follow-up appointments scheduled: n=16/21 (76%)  - Primary care provider (PCP) appointments scheduled: n=14/21 (67%)  - Both surgery follow-up and PCP appointments scheduled: n=12/21 (57%)  - Pharmacist-led medication reconciliation completed n=9/21 (43%)  - After hospital care plan delivered and taught: n=9/21 (43%)  - Post-discharge nursing call attempted: n=19/21 (90%)  - Post-discharge nursing call completed: n=16/21 (76%)  - Time from hospital discharge to nursing call: mean=2.32 days (SD=1.84) |
| Fisher et al., 2018; USA [44] | **Setting:** Subspecialty surgical services of colorectal surgery and surgical oncology at a tertiary care, academic medical hospital  **Surgery type:** Complex abdominal surgery: pancreatectomy, new ostomy patients, patients experiencing postoperative complications, or discharged with a drain in place. Extended to include: bowel perforations and emergent bowel resections, gastrectomy, tumour debulking with hyperthermic intraperitoneal chemotherapy | Assess context: Existing processes and resources listed  Changes to electronic systems: Button added to electronic system  Physical resources: Clinical space made available  Staffing changes and role clarification: New positions created and filled  Other implementation strategies: Not reported | - During the first month after implementation, there were inconsistencies regarding patient discharge instructions and teaching |
| Fitz et al., 2020; USA [45] | **Setting:** Academic medical centre  **Surgery type:** Lung transplantation | Assess context: Challenges identified reported  Select change management approach: Approach was selected  Other implementation strategies: Not reported | - Staff adherence to the discharge bundle process: ≥ 80% in the first month of the intervention  - Staff adherence to 2 of the 3 post-discharge phone calls within the first 7 business days after discharge: > 87% during all 3 months |
| Grahn et al., 2019; USA [46] | **Setting:** One university hospital and two community hospitals  **Surgery type:** Ileostomy | Not reported | - Intervention patients were more likely to have received telephone interaction from the clinic after hospital discharge: 90% vs 72%; *p* = 0.03 |
| Hu et al., 2020; China [30] | **Setting:** General tertiary level hospital  **Surgery type:** Kidney transplantation | Not reported | - Intervention fidelity not measured, however the authors used strategies to promote intervention fidelity |
| Iseler et al., 2018; USA [47] | **Setting:** Large, urban, healthcare system  **Surgery type:** Left ventricular assist device implantation | Staffing changes and role clarification: A staff member’s time was reallocated | - First home visit and clinic appointment scheduled as per protocol: n=8/10 (80%)  - For patients living in the outskirts of the 50-mile radius, scheduling this visit proved more challenging: n=2/10 (20%) |
| Koeckert et al., 2017; USA [48] | **Setting:** Department of cardiothoracic surgery  **Surgery type:** Cardiac; open and transcatheter valve | Assess context: Historical data provided | Not reported |
| Li et al., 2020; China [31] | **Setting:** Urology department in a hospital  **Surgery type:** Kidney transplantation | Staff education and training to support the intervention delivery: All staff passed the examinations  Other implementation strategies: Not reported | Not reported |
| Li et al., 2024; China [32] | **Setting:** Hospital  **Surgery type:** Colostomy | Staff education and training to support the intervention delivery: Nurses accomplished the training and assessment in ostomy-related courses | Not reported |
| Lin et al., 2024; China [34] | **Setting:** Tertiary hospital  **Surgery type:** Colostomy and ileostomy | Stakeholder engagement: 28 stakeholders determined facilitators and barriers to implementation of the discharge planning program  Staff education and training to support intervention delivery: Staff were “well-trained”  Other implementation strategies: Not reported | Not reported |
| Lin et al., 2025; China [33] | **Setting:** Grade 3A tertiary hospital  **Surgery type:** Colostomy | Staff education and training to support intervention delivery: Staff were “well-qualified” | Not reported |
| Liu et al., 2019; [27]  Robertson et al., 2018; USA  [26] | **Setting:** Department of neurosurgery, at one large, urban, quaternary care, academic, referral hospital project  **Surgery type:** Cranial or spinal neurosurgery | Not reported | Not reported |
| Mitchell, 2022; USA [49] | **Setting:** One orthopaedic unit at a tertiary hospital in urban city  **Surgery type:** Hip or knee joint replacement or revision | Determine project goals and measures of success: Clear goal set  Select change management approach: Approach was selected  Staffing changes and role clarification: Case manager lacked time and had issues with patient volume, influencing completion of intervention component. No coverage was provided when staff were absent  Other implementation strategies: Not reported | - Received a follow-up phone call: n=30 (100%); however, only n=14 (47%) received phone calls within the target 72-hour time frame  - Staff adherence with intervention components: 87% |
| Pelt et al., 2018; USA [50] | **Setting:** Tertiary referral centre  **Surgery type:** Total joint arthroplasty of hip or knee | Assess context: Historical data provided  Other implementation strategies: Not reported | Not reported |
| Tian et al., 2023; China [35] | **Setting:** Tertiary specialized women’s and children’s hospital  **Surgery type:** Vaginal natural orifice transluminal endoscopic | Not reported | Not explicit |
| Tseng et al., 2021; Taiwan [36] | **Setting:** Medical centre  **Surgery type:** Hip arthroplasty or internal fixation of hip fracture | Not reported | Adherence to rehabilitation in the home setting:  - First month: 36%  - First-third month: 36%  - Third-sixth month: 25%  - Sixth-twelfth month: 20% |
| Tu et al., 2024; China [37] | **Setting:** University hospital  **Surgery type:** Thoracic aortic endovascular repair | Not reported | - Intervention fidelity not measured, however the authors used strategies to promote intervention fidelity |
| Wang et al., 2025; China [38] | **Setting:** Tertiary hospital  **Surgery type:** Transurethral resection of prostate | Not reported | Not reported |
| Weintraub et al., 2018; USA [51] | **Setting:** Hospital  **Surgery type:** Coronary artery bypass graft | Changes to electronic systems: Software was expensive and difficult to implement. Requires ongoing modification  Other implementation strategies: Not reported | Not reported |
| Xu et al., 2021; China [39] | **Setting:** Orthopaedic department of a tertiary care hospital  **Surgery type:** Joint (hip or knee) replacement | Select change management approach: Approach was selected | Not reported |
| Yang et al., 2023; China [40] | **Setting:** Public teaching hospital  **Surgery type:** Percutaneous vertebroplasty | Not reported | - Completed 6 months follow up: 88%  - Did not start or complete treatment: 12% |
| Zhang et al., 2020; Zhang et al., 2021; China [28,29] | **Setting:** Medical centres of three large general hospitals  **Surgery type:** Permanent colostomy | Upskill people responsible for implementation process: All team members learnt the theory and care skills | Not reported |
| Zhou et al., 2023; China  [41] | **Setting:** 202 hospitals  **Surgery type:** Enterostomy | Staff education and training to support the intervention delivery: All passed the training and assessment | Not reported |
| Zuckerman et al., 2020; USA [52] | **Setting:** Eight institutions that were all existing members of the Quality Outcomes Database (a national registry that monitors spinal surgical care)  **Surgery type:** Lumbar fusion | Assess context: Registry and “drivers” data assessed and reported  Monitor process/outcomes: Not all patient data entered into electronic system limiting monitoring  Select change management approach: Approach was selected  Staffing changes and role clarification: Securing resources for staffing was a challenge  Other implementation strategies: Not reported | - All five discharge assessment questions asked: n=129 (62%) - Discharge plan existed: n=133 (69%) - Made post discharge phone call: n=159 (76%) |

USA, United States of America
